# Strength of Low-Frequency EEG Phase Entrainment to External Stimuli Is Associated with Fluctuations in the Brain's Internal State

**DOI:** 10.1523/ENEURO.0064-24.2024

**Published:** 2025-01-17

**Authors:** Verónica Mäki-Marttunen, Alexandra Velinov, Sander Nieuwenhuis

**Affiliations:** Cognitive Psychology Unit, Faculty of Social Sciences, Leiden University, Leiden, Alaska 2333, The Netherlands

**Keywords:** alpha power, arousal, EEG, entrainment, pupil

## Abstract

The brain attends to environmental rhythms by aligning the phase of internal oscillations. However, the factors underlying fluctuations in the strength of this phase entrainment remain largely unknown. In the present study, we examined whether the strength of low-frequency electroencephalography (EEG) phase entrainment to rhythmic stimulus sequences varied with the pupil size and posterior alpha-band power, thought to reflect the arousal level and excitability of posterior cortical brain areas, respectively. We recorded the pupil size and scalp EEG while participants carried out an intermodal selective attention task, in which they were instructed to attend to a rhythmic sequence of visual or auditory stimuli and ignore the other perceptual modality. As expected, intertrial phase coherence (ITC), a measure of entrainment strength, was larger for the task-relevant than for the task-irrelevant modality. Across the experiment, the pupil size and posterior alpha power were strongly linked with each other. Interestingly, ITC tracked both variables: larger pupil size was associated with a selective increase in entrainment to the task-relevant stimulus sequence, whereas larger posterior alpha power was associated with a decrease in phase entrainment to both the task-relevant and task-irrelevant stimulus sequences. Exploratory analyses showed that a temporal relation between ITC and posterior alpha power emerged in the time periods around pupil maxima and pupil minima. These results indicate that endogenous sources contribute distinctly to the fluctuations of EEG phase entrainment.

## Significance Statement

Fluctuations in cortical state powerfully shape the perception of external stimuli. Understanding the physiological signatures of cortical state fluctuations is crucial to understand how the brain selectively attends and switches between internal and external content. We studied how two signatures of attentional state, pupil-linked arousal and power in the alpha band, shape the entrainment of brain activity to low-frequency rhythmic stimuli. Our results reveal common and dissociable influences of these signatures at slow time scales. Furthermore, measuring and including the pupil size and posterior alpha power as covariates in statistical models can help increase statistical power in studies focusing on electroencephalography phase entrainment. Our study provides new evidence on a direct influence of cortical state on the perception of rhythmic stimuli.

## Introduction

How we perceive the continuous stream of external sensory stimulation is highly modulated by the internal state of the brain (Mathewson et al., 2012), but the interaction between external and internal mechanisms of attention is still poorly understood. Rhythmic stimuli entrain endogenous oscillatory activity in the brain to the temporal structure of the stimulus sequence (VanRullen et al., 2014; Ten Oever et al., 2017; Zoefel et al., 2018; Bauer et al., 2021). This “neural entrainment” results in the alignment of the high-excitability oscillatory phase with the predicted onset of the rhythmic stimuli (Lakatos et al., 2008; Stefanics et al., 2010) and provides an advantage for the processing of natural stimuli that are inherently rhythmical, such as speech, music, and repetitive motion (Henry and Obleser, 2012; Lakatos et al., 2019). Although phase alignment of oscillations to external rhythms constitutes an important principle of neural dynamics and attention, little is known about the factors that make the strength of phase entrainment fluctuate over time.

Here, we investigated the impact on low-frequency phase entrainment of two physiological measures known to be highly variable across time: pupil-linked arousal and posterior alpha-band power. Attention during tasks requiring sustained performance commonly fluctuates due to variations in the internal state of vigilance or arousal (Unsworth and Robison, 2017). The pupil size has long been used to measure variations in the arousal level in humans (Bradshaw, 1967). Very low or very high levels of vigilance, as characterized by lapses of attention or task-unrelated thoughts, often occur during periods of small and/or large (pretrial) baseline pupil diameter (Unsworth and Robison, 2016; van den Brink et al., 2016; Madore et al., 2020). Whether and how arousal modulates the strength of neural entrainment, which is an externally directed process, is still unknown.

Changes in posterior alpha-band power, as measured with electroencephalography (EEG), are thought to reflect the excitability of posterior cortical brain areas that process external (sensory) input (Romei et al., 2008; Samaha et al., 2017). Increased alpha power is suggested to facilitate internally directed attention rather than attention to external stimuli (Cooper et al., 2003; Klimesch et al., 2007; Benedek et al., 2014). Lapses of attention and task-unrelated thoughts are associated with increases in pretrial posterior alpha power (O'Connell et al., 2009; Compton et al., 2019; Jin et al., 2019; Madore et al., 2020; Groot et al., 2021). Similarly, a study in nonhuman primates found that periods with high alpha power were characterized by attentional lapses and strongly diminished low-frequency entrainment of neuronal oscillations to task-relevant rhythmic stimulus sequences (Lakatos et al., 2016). These results suggest that alpha power is an index of internal state and would be expected to anti-correlate with the strength of entrainment to a task-relevant sequence of stimuli.

Although the baseline pupil size and posterior alpha have both been associated with attentional state, their interrelation may not be straightforward. Alpha power as a measure of inattention, as reviewed above, would be expected to correlate negatively with pupil size. That is, low arousal would be related to distraction and an increase in the “idling” alpha. However, studies on the relationship between pupil size and posterior alpha power at rest or in pretrial periods have found a positive relation and/or an inverted-U relationship (Hong et al., 2014; van Kempen et al., 2019; Ceh et al., 2020; Podvalny et al., 2021; Pfeffer et al., 2022; Waschke et al., 2019). Therefore, these two signals may reflect brain states that are at least partly dissociable, and we expected that they would distinctly influence neural entrainment.

In this study, we aimed to test whether and how low-frequency phase entrainment is susceptible to modulations in internal state, as indexed by pupil-linked arousal and posterior alpha power. We simultaneously recorded the pupil size and scalp EEG to investigate the relationships between pupil-linked arousal, cortical alpha oscillations, and low-frequency phase entrainment during an intermodal attention task. In the task, rhythmic streams of visual and auditory stimuli were concurrently presented. Participants had to detect infrequent targets in the cued modality and ignore the other modality. A small difference in the repetition rate (visual, 1.1 Hz; auditory, 1.4 Hz) allowed us to separately assess entrainment to the task-relevant and task-irrelevant stimulus sequences. We hypothesized that transient changes in the strength of entrainment to the attended modality would be related to fluctuations in the arousal level of the participants. We also expected that internal neural processes signaled by alpha power would modulate the entrainment level. This study brings novel insights into the interrelation between external and internal attentional mechanisms of the brain.

## Materials and Methods

### Participants

Thirty-seven students recruited at Leiden University took part in the experimental study. One participant was excluded because the recording was interrupted. EEG data from four participants were excluded from analysis due to excessive artifacts (>50% of epochs rejected) in the attend visual condition. One participant was excluded because of low accuracy (hit rate < 0.5) in the attend auditory condition. Five out of the first 15 participants were excluded because they reported an unforeseen visual illusion, where they perceived the afterimage of the green fixation stimulus having a similar color as the magenta targets, probably caused by a complementary afterimage effect (Manzotti, 2017). As a precaution to avoid this illusion, after the first 15 participants, we changed the color stimulus, as reported below. Performance did not differ between either set of stimuli for the participants included in the final study (hit rate, first round, 0.77 ± 0.05; second round, 0.79 ± 0.07; *T* = 0.86; *p* = 0.398; incorrect responses, first round, 32 ± 30; second round, 38 ± 38; *T* = 0.41; *p* = 0.685). The final sample consisted of 26 participants (mean age, 23 years; age range, 18–28 years; 22 females). Study exclusion criteria included any psychiatric disorders and wearing contact lenses. The experimental protocol was reviewed and approved by the ethics committee of Leiden University. Participants signed a consent form prior to participating and received a compensation of 7.5 euros/hour or course credits.

### Task and stimuli

**Figure 1.**
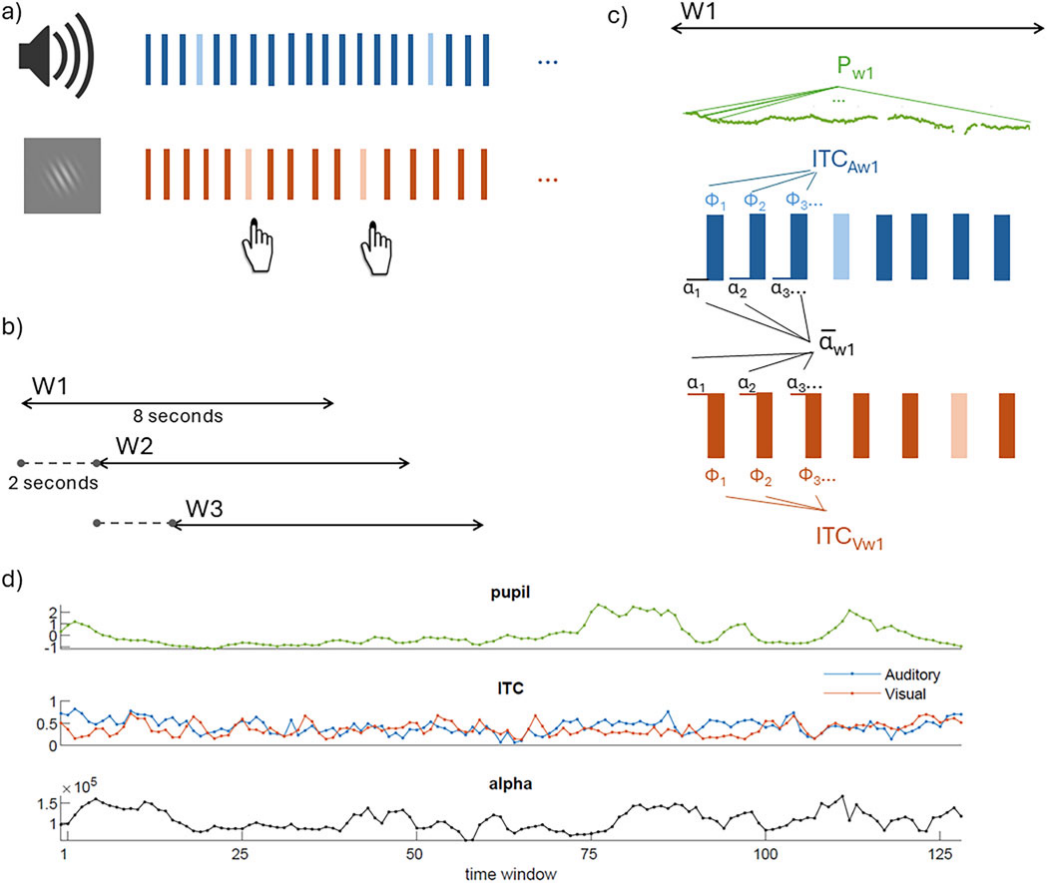
Task and analysis overview. ***a***, Multimodal task. Toy example of a sequence of stimuli sequences of the intermodal selective attention task. Rectangles represent stimuli of the auditory modality (tones, represented in blue) and the visual modality (Gabor patches, represented in orange). Auditory and visual stimuli were presented simultaneously at slightly different frequencies (auditory, 1.4 Hz; visual, 1.1 Hz). Participants had to attend to only one modality per block and respond to the target stimuli in that modality (represented in lighter color) with a button press. In this example, the participant responds to the target stimulus in the visual modality—i.e., the visual stimuli were task-relevant. ***b***, Illustration of sliding windows approach. The pupil size, ITC, and alpha power were calculated in 8 s sliding windows (W1, W2, W3, etc.) covering the whole block, with 2 s steps. ***c***, Illustration of the calculation of ITC over auditory (ITC_A_) and visual (ITC_V_) stimuli types in one 8 s sliding window. The average pupil size (P, pupil trace in green) was calculated for the entire window. Alpha power for each window was calculated as the average prestimulus alpha power across all stimuli. ITC represented the coherence of phase values at a given frequency across all the trials of the same modality. ***d***, A participant's single-block sample data of time courses of the various measures, where each observation represents one time window. These time courses were used to study the relation between pupil, prestimulus alpha power, and ITC.

Participants performed an intermodal selective attention task (Fig. 1), based on that used by Lakatos et al. (2016) to study phase entrainment in monkeys. Participants were presented with simultaneous streams of visual and auditory stimuli and had to respond as quickly as possible to rare target stimuli in the cued modality by pressing the “B” key on a keyboard and to otherwise withhold responding. The response deadline was 1 s after the target onset. The task consisted of 10 blocks, each lasting ∼5 min. The participants were prompted at the beginning of each block to attend to the visual stimuli (attend visual condition) or to the auditory stimuli (attend auditory condition) and ignore the other modality. Half of the blocks required the participant to attend to the visual modality and the other half to the auditory modality in a quasirandom order (A1-V1-A2-A3-V2-A4-V3-V4-A5-V5).

In each block, 605 stimuli were presented: 265 visual stimuli and 340 auditory stimuli. In each stream of stimuli, 15% of the stimuli were targets and 85% were standards. The auditory stimuli consisted of short beeps with a 460 Hz pitch for the standards and a 476 Hz pitch for the targets. The visual stimuli consisted of colored RGB Gabor patches (orange standards, RGB 229 133 126; magenta targets, RGB 229 90 172; green fixation point for participants #1-15, RGB 151 199 62; purple fixation point for participants #16-37, RGB 180 137 205) with a 10° orientation and a frequency of 0.1 cycles/pixel, created using an online Gabor-patch generator (https://www.cogsci.nl/gabor-generator). Because changes in luminosity can drive changes in the pupil size, we made sure that the visual stimuli and the interstimuli screens were isoluminant. For this, visual stimuli were made isoluminant with the gray screen background (RGB 167 167 167) using the SHINEcolor toolbox (Dal Ben, 2023). The visual and auditory stimuli were presented at average frequencies of 1.1 and 1.4 Hz, respectively. All stimuli were presented for 50 ms. The fixation point was on the screen in the interval between consecutive visual stimuli, and all visual stimuli subtended a 4° angle on the screen. The average interstimulus interval was 387 ms, with a range from 0 (i.e., auditory and visual stimuli presented simultaneously) to 727 ms. To lower a possible contribution of stimulus-evoked EEG responses to our measure of intertrial phase coherence (ITC), we added to the interstimulus interval some decorrelated jitter, drawn on each stimulus time from the values [−66, −33, 0, 33, 66 ms]. Entrainment occurs at the average (i.e., expected) frequency of presentation of the stimuli and thus is independent of the stimulus presentation itself (Besle et al., 2011).

### Procedure

Participants were instructed to sit as still as possible throughout the blocks and to always look at the center of the screen, also in the attend auditory condition. Their chins were positioned in a chin rest, with their eyes at ∼73 cm from a monitor with a refresh rate of 60 Hz. For the auditory stimuli, custom in-ear air–tube headphones were used to prevent electrical artifacts in the EEG recordings. Participants first performed two practice blocks, one for each condition, which each lasted 2 min and contained a total of 256 visual and auditory stimuli. After the practice blocks, the eye-tracker was calibrated, and the participants performed the task in a dimly lit room (19.9 lx). The task was controlled using Eprime 3.0 with the appropriate Tobii extensions installed. In between blocks, participants could take breaks and decide when to start the following block. Lighting conditions and screen luminance were kept constant across the whole session. Participants took between 60 and 70 min to complete the experiment.

### Time window approach

The purpose of this study was to examine the slow temporal relation between pupil-linked arousal, entrainment, and alpha power. Based on a previous study (Lakatos et al., 2016), we analyzed the pupil and EEG data in sliding time windows. The windows had a length of 8 s and a step size of 2 s, resulting in an overlap of 6 s between consecutive time windows (Fig. 1). The window length and step size were chosen to achieve a balance between the number of stimuli included in each estimate of entrainment (larger number provides a better estimate) and the temporal resolution of our analyses (shorter window length provides higher resolution). Each time window included ∼9 visual stimuli and ∼11 auditory stimuli.

Given that the paradigm involved the simultaneous presentation of visual and auditory stimuli, each time window contained stimuli in both modalities, but depending on the block, only one of them was task-relevant. For each time window, we obtained the following five measures (Fig. 1): (1) an average pupil size across the entire 8 s window; ITC (a measure of entrainment), which was calculated based on the phase angles at stimulus onset of all (2) visual stimuli and all (3) auditory stimuli of which the onset latency fell within the 8 s window; and average prestimulus alpha power across all (4) visual stimuli and all (5) auditory stimuli of which the onset latency fell within the 8 s window.

Note that in our key analyses, we recoded the visual and auditory stimulus sequences as “task-relevant” and “task-irrelevant,” based on the participant’s instruction, so that for each 8 s window, we had task-relevant and task-irrelevant ITC and prestimulus alpha power values.

Below, we explain how the pupil size, ITC, and prestimulus alpha power were computed.

### Pupillometry

The pupil diameter of both eyes was recorded using a Tobii Pro X3-120 eye-tracker with a sampling rate of 40 Hz. The calibration was performed at the beginning of the task using a five-point fixation procedure. The Tobii device automatically marked periods when no pupil signal was recorded (e.g., blinks), and the custom code was compiled to save accurate timing data that could then be used to create eye-tracking epochs and segmentations for the behavioral data. Raw pupil data were preprocessed using the PhysioData Toolbox v0.5.0 (Kret and Sjak-Shie, 2019) using the standard pipeline of the Pupil Diameter Analyzer module. One participant was excluded from the pupil analyses due to excessive data loss during preprocessing (>50% of recording). An exponential decay in the pupil size was observed at the beginning of the blocks for most participants. Since the magnitude of such time-on-task effects can obscure the effects of the more subtle trial-by-trial fluctuations in pupil-linked arousal that we were interested in (van den Brink et al., 2016), we fitted an exponential decay curve to the pupil time series using the MATLAB R2018 (MathWorks) functions *fittype* and *fit* and regressed out this component from the pupil data. For the time windows analyses, the continuous pupil signal was cut into 8 s windows (2 s step size) and then averaged across each window. The resulting time series were *z*-scored separately for each block to make sure that differences in the pupil size reflected local fluctuations in the pupil size instead of global time-on-task effects (Hopstaken et al., 2015).

### EEG acquisition and preprocessing

EEG data were recorded using a BioSemi ActiveTwo system with 32 active Ag-AgCl electrodes, at a sampling rate of 512 Hz. The electrodes were arranged according to the international 10–20 system. In addition to the 32 main electrodes, two reference electrodes were positioned on the mastoids, two facial electrodes above and below the left eye, and two electrodes on the temples. Raw EEG data were preprocessed in EEGLAB (Delorme and Makeig 2004). The signal was re-referenced to the mastoids and high-pass filtered (0.5 Hz). Bad channels were removed using *cleanrawdata* function of EEGLAB. The minimal acceptable correlation with nearby channels was set to the default value of 0.8, while the parameter for artifact subspace reconstruction (a method for removing high-amplitude artifacts from EEG data) was set to 10. Missing channels were interpolated from neighboring sites. Then the data were split into attend auditory and attend visual conditions and epoched into 3 s segments centered on the stimulus onset. This epoch length was chosen to avoid edge artifacts in the time–frequency analysis (Besle et al., 2011). Bad epochs were rejected automatically (threshold criteria, 150 μV), and an independent component analysis (infomax algorithm with “extended” option to extract sub-Gaussian sources) was performed to identify and remove noise components manually (e.g., eye artifacts, muscle artifacts). Preprocessing was done using the Neuroscience Gateway (https://www.nsgportal.org, Sivagnanam et al., 2013; Martínez-Cancino et al., 2021).

### ITC and prestimulus alpha power

The epoched EEG data were used to calculate measures of phase entrainment and alpha power. We first extracted spectral power and phase dynamics using Morlet wavelet decomposition from 1 to 30 Hz with 30 logarithmically spaced steps. We used a wavelet cycle range of 3–12. This corresponded to a precision, as measured by the spectral full-width at half-maximum, ranging from 0.48 to 5.88 Hz (Cohen, 2019). Entrainment of EEG oscillations to the stimulus rhythms is calculated based on the instantaneous phase values at the frequency and time of presentation of visual and auditory stimuli (Besle et al., 2011; Lakatos et al. 2013). To determine the phase angle at the stimulus onset, we used the 3 s epochs centered on the stimulus onset that were mentioned above (section “EEG acquisition and preprocessing”). Our ITC estimates based on the instantaneous phase at *t* = 0 were not affected by edge-effect artifacts (i.e., were within the cone of influence). ITC, a measure of the consistency across trials of the phase angle at the time of the stimulus onset (Tallon-Baudry et al., 1996), was calculated with the following formula:

$$\mathrm{ITC}(\;f,t)\;=\;\mathrm{abs}(\mathrm{sum}(e^{i*\mathrm{phi}(\;f,t)})/n),$$

where phi is the phase at frequency *f* and time point *t* of a single trial (i.e., stimulus presentation) and *n* is the number of trials considered. This was done separately for the visual and auditory stimulus streams at the corresponding frequency (1.1 Hz for visual and 1.4 Hz for auditory; Fig. 3*a*). For these analyses, we averaged data from electrodes displaying the largest ITC for each modality: AF3, AF4, F3, Fz, F4, FC1, and FC2 for auditory stimuli and C3, Cz, C4, CP1, and CP2 for visual stimuli. For the 8 s time window analyses, we computed the ITC across all visual and (separately) auditory stimuli of which the onset latency fell within the 8 s window.

For the analysis of prestimulus alpha power, we extracted for each epoch the data from −500 to 0 msec before the stimulus and applied a fast Fourier transform to extract the power density spectrum (Ergenoglu et al., 2004; Compton et al., 2019). We then calculated the area under the curve for the alpha band (8–13 Hz). Extended Data Figure 3-1 shows the posterior distribution of prestimulus alpha power. We averaged the data of posterior electrodes (P7, P3, Pz, O1, Oz, O2, P4, and P8), obtaining thus one prestimulus alpha value for each stimulus. For the 8 s time window analyses, we computed the average alpha power value across all visual and auditory stimuli of which the onset time fell within the 8 s window. The resulting time series of ITC and alpha power were *z*-scored separately for each block.

The analyses described above eventually yielded one pupil size value for each 8 s window and two ITC values and two alpha power values for each 8 s window: one for the task-relevant stimulus sequence (visual stimuli in the attend visual condition; auditory stimuli in the attend auditory condition) and one for the task-irrelevant stimulus sequence (visual stimuli in the attend auditory condition; auditory stimuli in the attend visual condition).

### Temporal analysis around pupil maxima and minima

We analyzed the variables ITC and prestimulus alpha power in segments of 10 windows around maxima and minima of the pupil timecourse. Because of the overlap between windows, 10 windows corresponded to 26 s. The normalized pupil size timecourse of each participant was thresholded at +1 standard deviation to detect the peaks and −1 standard deviation to detect the troughs. For this, the function *findpeaks* in MATLAB was employed, with a minimum distance between consecutive maxima and consecutive minima of 10 time windows. Then, the normalized ITC and alpha power data were extracted from those windows for further analysis. The cross-correlation between the variables was calculated with the function *xcorr* in MATLAB. To statistically compare cross-correlations between the time-windowed variables around pupil maxima and minima, we used cluster-based permutation testing, with 1,000 iterations and a significance level of *α* = 0.01 for the individual time points and *α* = 0.05 for the significant clusters. All the temporal analyses were performed on the task-relevant modality, and data were collapsed across modalities.

### Statistics

The hit rate was defined as the proportion of targets in the cued modality that were detected in time (i.e., button press within 1 s after the target onset). Any response that was not a hit was counted as an incorrect response. Given the overlap between the task-relevant and task-irrelevant stimulus sequences, it was hard to distinguish incorrect responses to standards in the task-relevant modality from responses to the targets of the task-irrelevant modality, and thus we pooled them together as “incorrect responses.” Average ITC values were compared with repeated-measure ANOVA with cued modality (attend visual, attend auditory) and stimulus type (visual, auditory) as within-subject factors. To examine the relationships between the pupil size, ITC, prestimulus alpha power, and task performance (hit rate, number of incorrect responses), we aggregated the data of the 8 s time windows in 10 equally populated bins on the basis of one variable and examined the effects of bin (1–10), task relevance (task-relevant vs task-irrelevant stimulus sequence), and stimulus type (visual, auditory) on one of the other variables using a repeated-measure ANOVA. In the analyses of task performance, we included in the model both a linear and quadratic effect of bin. Statistics were performed using MATLAB.

## Results

### Behavioral results

**Figure 2.**
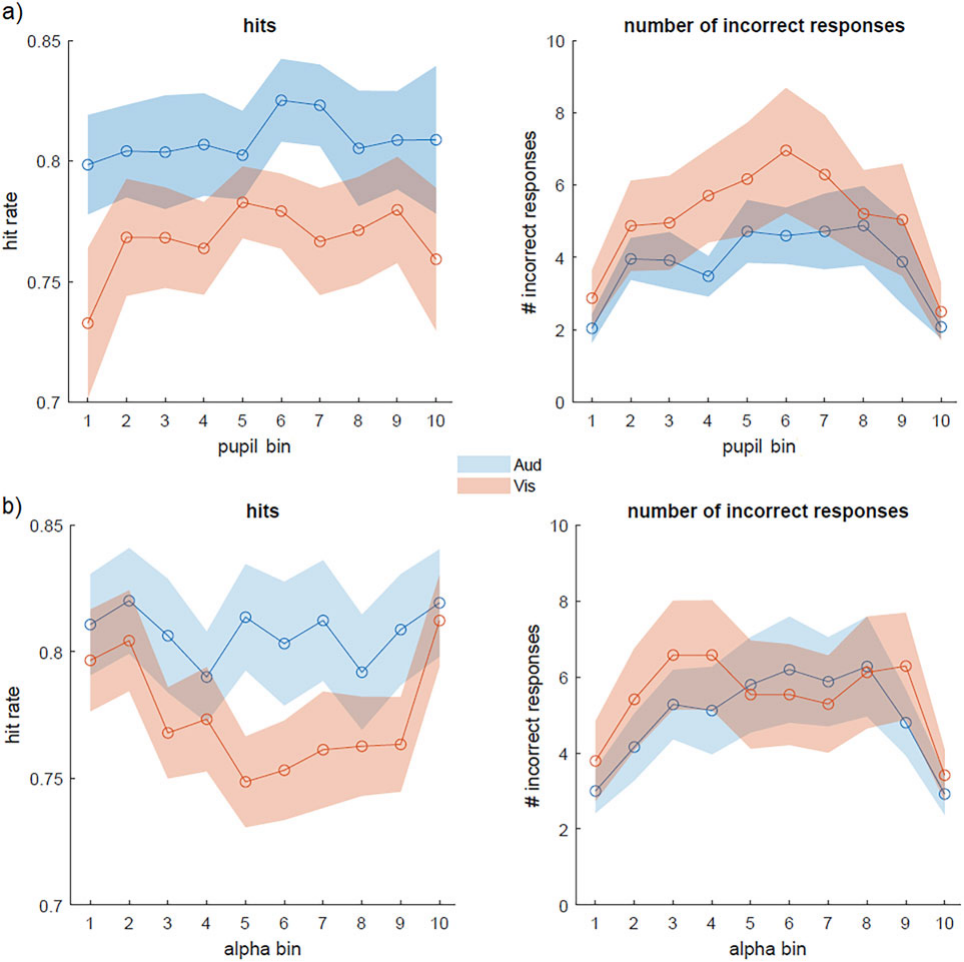
Behavioral results. The hit rate and number of incorrect responses as a function of pupil bin (***a***) and prestimulus alpha bin (***b)***. Colors correspond to the different task-relevant modalities (Aud, auditory; Vis, visual).

Participants showed better performance in the attend auditory condition than in the attend visual condition (Fig. 2*a*). The hit rate was significantly higher (*F*_(1,495)_ = 6.78; *p *= 0.009), whereas the number of incorrect responses did not differ between conditions (*F*_(1,475)_ = −0.88; *p *= 0.11; for statistics, Table 1). We also inspected the relation between the pupil size and task performance. Pupil bin had no linear and quadratic effects on the hit rate (*F*_(1,495)_ = 1.79; *p *= 0.18; *F*_(1,495)_ = 2.52; *p *= 0.11). In contrast, pupil bin had both a linear and a quadratic effect on the number of incorrect responses (*F*_(1,475)_ = 22.65; *p *< 0.001; *F*_(1,475)_ = 30.90; *p *< 0.001). More incorrect responses were made at intermediate levels of the pupil size (Fig. 2*a*). No significant interactions between pupil size bin and cued modality were observed (hits, *p *= 0.64; incorrect responses, *p *= 0.74).

**Table 1.** Statistical table

	Data structure	Type of test	*p* value	Power (95% C.I., min, max)
Figure 2*a*	Normal distribution	Repeated-measure ANOVA: pupil bin effect	0.92	−0.004, 0.025
	Normal distribution	Repeated-measure ANOVA: bin quadratic effect	0.11	−0.001, 0.0002
	Normal distribution	Repeated-measure ANOVA: Modality effect	0.009	−0.08, −0.011
	Normal distribution	Repeated-measure ANOVA: pupil bin effect	<0.001	1.00, 2.41
	Normal distribution	Repeated-measure ANOVA: bin quadratic effect	<0.001	−0.19, −0.09
	Normal distribution	Repeated-measure ANOVA: modality effect	0.1	−0.28, 2.97
Figure 2*b*	Normal distribution	Repeated-measure ANOVA: prestimulus alpha bin effect	0.62	−0.01, 0.01
	Normal distribution	Repeated-measure ANOVA: bin quadratic effect	0.76	−0.001, 0.0008
	Normal distribution	Repeated-measure ANOVA: modality effect	0.14	−0.05, 0.008
	Normal distribution	Repeated-measure ANOVA: prestimulus alpha bin effect	<0.001	0.80, 2.39
	Normal distribution	Repeated-measure ANOVA: bin quadratic effect	<0.001	−0.18, −0.07
	Normal distribution	Repeated-measure ANOVA: modality effect	0.05	−0.06, 3.59
Figure 3*c*	Normal distribution	Repeated-measure ANOVA: task-relevance effect	0.006	−0.05, 0.00
	Normal distribution	Repeated-measure ANOVA: modality effect	<0.001	0.05–0.12
Figure 4*a*	Normal distribution	Repeated-measure ANOVA: pupil bin effect	0.68	−0.002, 0.003
	Normal distribution	Repeated-measure ANOVA: modality effect	0.844	−0.022, 0.018
	Normal distribution	Repeated-measure ANOVA: attention effect	0.011	−0.046, −0.005
	Normal distribution	Repeated-measure ANOVA: interaction bin * attention	0.005	0.001, 0.007
Figure 4*b*	Normal distribution	Repeated-measure ANOVA: pupil bin effect	<0.001	0.015, 0.023
	Normal distribution	Repeated-measure ANOVA: modality effect	0.939	−0.010, 0.010
Figure 4*c*	Normal distribution	Repeated-measures ANOVA: prestimulus alpha bin effect	0.002	−0.007, −0.001
	Normal distribution	Repeated-measure ANOVA: Modality effect	0.058	−0.037, 0.000
	Normal distribution	Repeated-measure ANOVA: attention effect	0.515	−0.012, 0.025
	Normal distribution	Repeated-measure ANOVA: interaction bin * attention	0.445	−0.001, 0.004

Prestimulus alpha power showed a qualitatively similar relationship with task performance. Alpha bin had no linear and quadratic effects on the hit rate (*F*_(1,515)_ = 0.04; *p *= 0.62; *F*_(1,515)_ = 0.08; *p *= 0.76). In contrast, alpha bin had both a linear and a quadratic effect on the number of incorrect responses (*F*_(1,495)_ = 15.678; *p *< 0.001; *F*_(1,495)_ = 19.135; *p *< 0.001). More incorrect responses were made at intermediate levels of prestimulus alpha power (Fig. 2*b*). No significant interaction between alpha power bin and cued modality was observed (hits, *p *= 0.16; incorrect responses, *p *= 0.89).

### Low-frequency phase entrainment to simultaneous trains of auditory and visual stimuli

**Figure 3.**
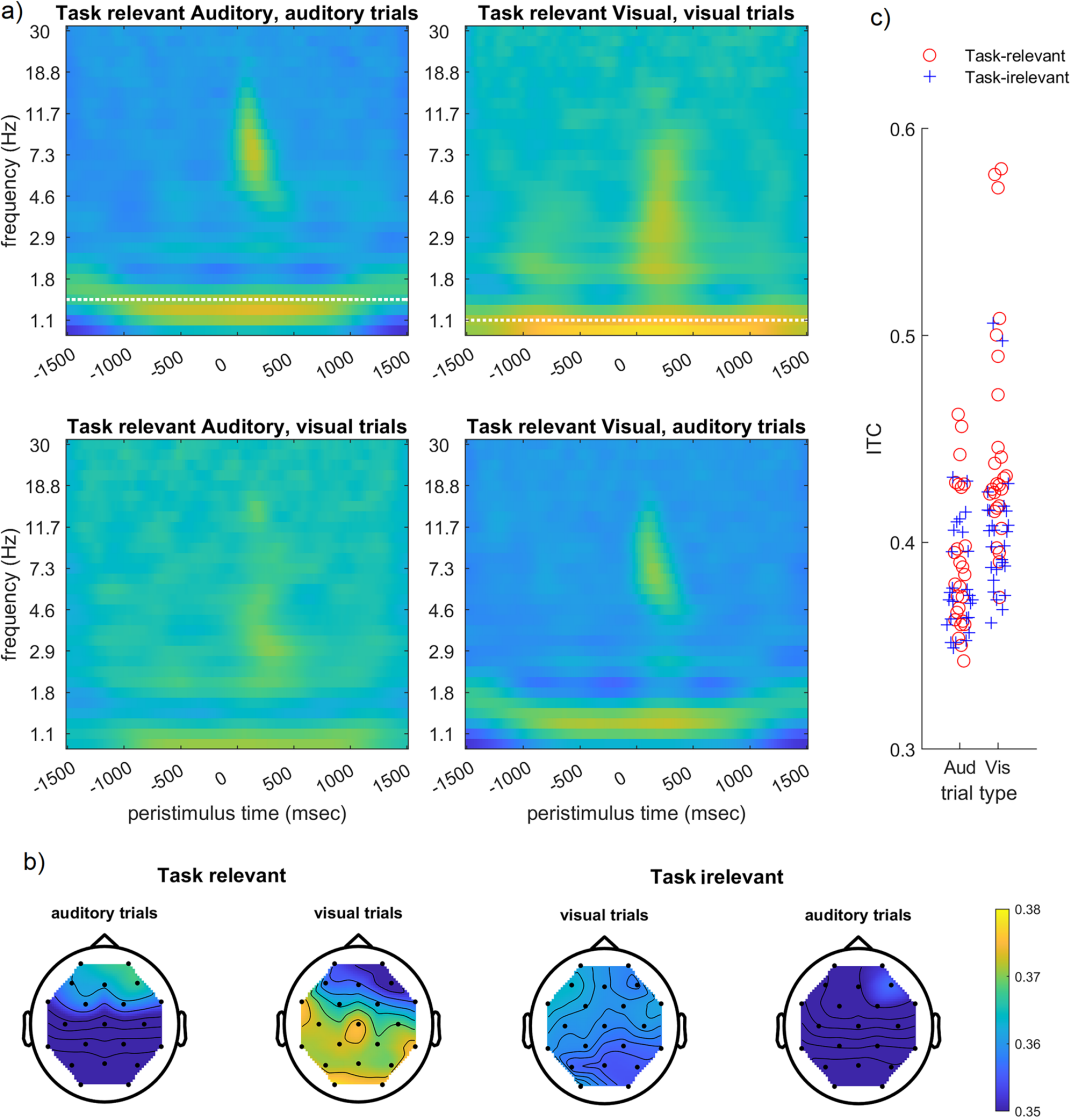
Low-frequency phase entrainment. ***a***, ITC time–frequency spectra, averaged across all electrodes. White horizontal lines indicate the average presentation frequency of auditory (top left) and visual (top right) stimuli. ***b***, Topographies of ITC strength at the peak frequency and the time of the stimulus onset. Topoplots for alpha power are displayed in Extended Data Figure 3-1. ***c***, Average ITC at the driving frequency as a function of task relevance (task-relevant vs task-irrelevant stimulus sequence) and stimulus type.

Figure 3*a* shows the ITC spectra for the various conditions. The top row shows strong ITC at the frequency corresponding to the task-relevant stimulus sequence (1.4 Hz for attend auditory; 1.1 Hz for attend visual), suggesting entrainment of EEG oscillations to the task-relevant stimulus rhythm. The corresponding ITC topography was frontal for the auditory stimulus sequence and central for the visual stimulus sequence (Fig. 3*b*). The bottom row of Figure 3*a* suggests that EEG oscillations also became entrained to the task-irrelevant stimulus sequences, although significantly less than the task-relevant stimulus sequences (Fig. 3*c*) and with more distributed topographies (Fig. 3*b*). The ITC associated with the task-relevant stimulus sequence was larger in the visual than in the auditory modality (*F*_(1,100)_ = 28.67; *p* < 0.001; Fig. 3*c*), and the difference between entrainment to task-relevant and task-irrelevant stimulus sequences was also larger in the visual modality (interaction task relevance * stimulus type, *F*_(1,100)_ = 7.68; *p* = 0.006; task-relevant vs task-irrelevant, visual, *p* < 0.001; auditory, *p* = 0.056).

10.1523/ENEURO.0064-24.2024.f3-1 Figure 3-1 Topoplots of pre-stimulus alpha power distribution for task-relevant stimuli. Download Figure 3-1, TIF file.

### Relation between the pupil size, ITC, and prestimulus alpha power

**Figure 4.**
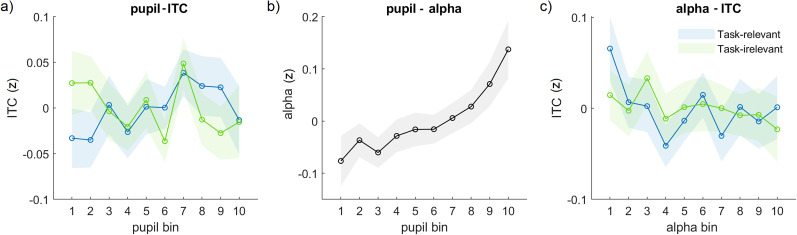
Paired relation between the pupil size, ITC, and alpha power. Relation between (***a***) the pupil size and ITC; (***b***) the pupil size and prestimulus alpha power; and (***c***) prestimulus alpha power and ITC. Different colors correspond to the task-relevant and task-irrelevant modality. Note that the distinction between the task-relevant and task-irrelevant modality is only of interest for ITC. There were no significant main effects and interaction effects of the stimulus type (visual, auditory; Extended Data Fig. 4-1), so we averaged across those levels. The significant effects cannot be explained by the number of targets (Extended Data Fig. 4-2).

We next inspected the paired associations between the pupil size, ITC, and prestimulus alpha power. ITC did not show a main effect of pupil bin (Fig. 4*a*; *F*_(1,992)_ = 0.16; *p* = 0.68). However, there was a significant interaction between bin and task relevance (*F*_(1,992)_ = 7.74; *p* = 0.005). Post hoc linear regression analyses showed that with increasing pupil size, the low-frequency oscillations became more entrained to the task-relevant stimulus sequence (*β* = 0.005; *t* = 2.22; *p* = 0.026), while the numerical decrease in entrainment to the task-irrelevant stimulus sequence was nonsignificant (*β* = −0.003; *t* = −1.70; *p* = 0.089).

10.1523/ENEURO.0064-24.2024.f4-1 Figure 4-1 Paired relationships between pupil size, ITC and alpha power separating by stimulus modality. Left column: auditory stimuli in Attend Auditory blocks (i.e., auditory stimuli were task-relevant, blue) and Attend Visual blocks (i.e., auditory stimuli were task-irrelevant, green). Right column: visual stimuli in Attend Visual blocks (i.e., visual stimuli were task-relevant, blue) and Attend Auditory blocks (i.e., visual stimuli were task-irrelevant, green). Top row: pupil size and ITC; Middle row: pupil size and prestimulus alpha power; bottom row: prestimulus alpha power and ITC. Download Figure 4-1, TIF file.

10.1523/ENEURO.0064-24.2024.f4-2 Figure 4-2 Effect of number of targets on pupil size, ITC and alpha power. We examined if the relationships between our four key dependent variables might be confounded by enlarged evoked EEG or pupil responses to infrequent target stimuli. The temporal overlap between the auditory and visual sequences made it hard to estimate single-trial evoked responses, but we used a more indirect way to address the issue: We assessed if ITC, and average alpha power and pupil size in a given window depended on the number of targets in that window. If two variables were each dependent on the number of targets, then their relationship might be due to this confound rather than slow co-fluctuations in internal state. We found no effect of number of targets (0, 1 or 2+) on ITC in the task-relevant (F = 1.35, p = 0.25) or task-irrelevant modality (F = 0.99, p = 0.41, Fig. 4.2). We found no effect of number of targets on prestimulus alpha (F = 1.47, p = 0.228). We found an effect of number of targets on pupil size (F = 2.91, p = 0.020), with increasing pupil size for increasing numbers of targets. The absence of an effect on ITC and alpha power suggests that number of targets cannot be driving the significant relation between this variable and pupil size in Fig. 4.b. Download Figure 4-2, TIF file.

When looking at prestimulus alpha power, we found a highly significant main effect of pupil bin on alpha power (Fig. 4*b*; *F*_(1,998)_ = 108.27; *p* < 0.001). Prestimulus alpha power directly increased with pupil size, in line with a previous study (Podvalny et al., 2021). Furthermore, ITC showed a significant main effect of alpha power bin (Fig. 4*c*; *F*_(1,1032)_ = 9.00; *p* = 0.002), with decreasing ITC as alpha power increased (*β* = −0.004). The interaction with task relevance was not significant (*F*_(1,1032)_ = 0.58; *p* = 0.445).

We did not find any significant main effect of the stimulus type (auditory vs visual) or interaction between the stimulus type and pupil/alpha bin (*p *> 0.1; Extended Data Fig. 4-1), suggesting that the key results reported here generalized across perceptual modalities. Also, our key results could not be explained in terms of enlarged evoked EEG or pupil responses to infrequent target stimuli (Extended Data Fig. 4-2).

### Arousal-related modulation of the temporal relation between ITC and alpha power

**Figure 5.**
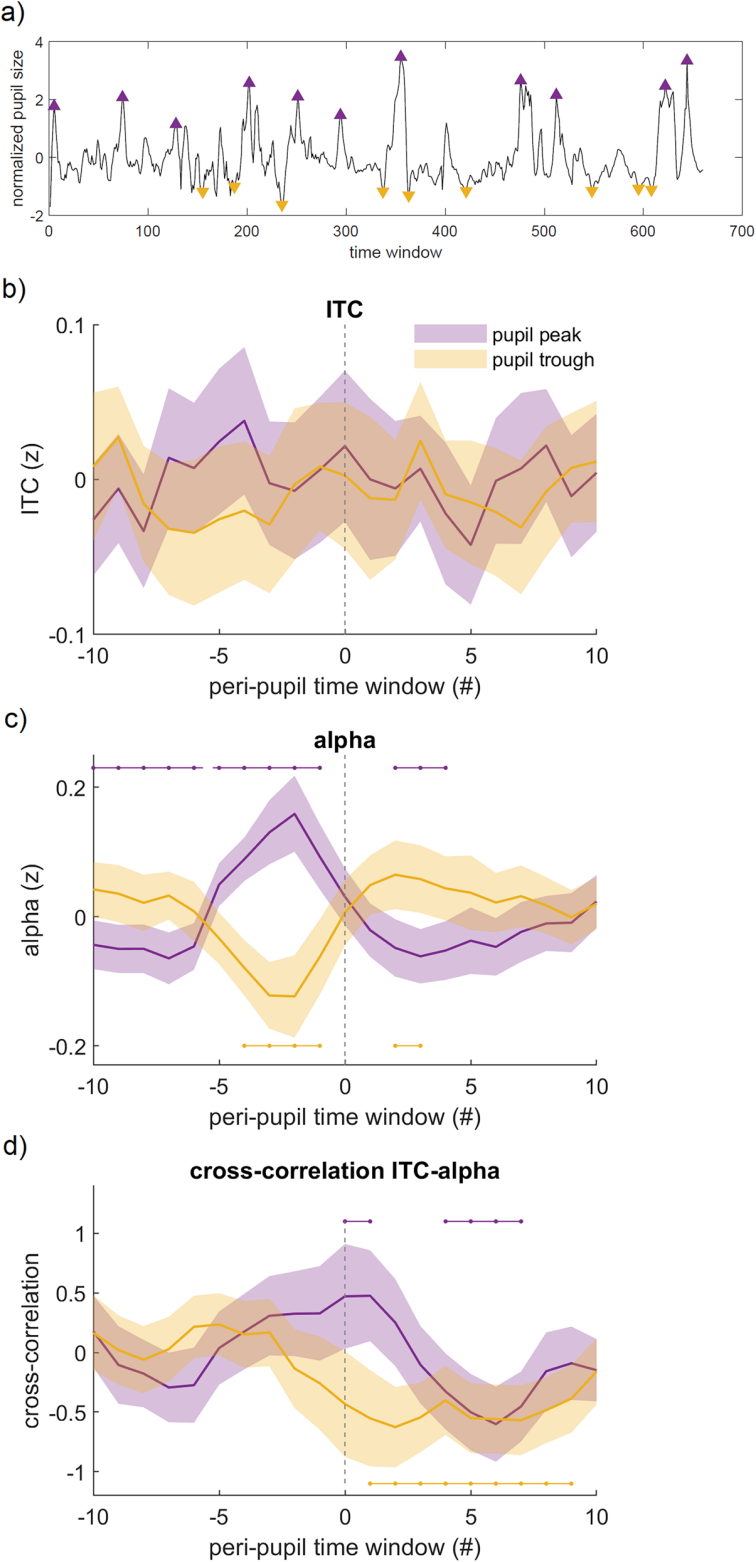
Temporal relation between ITC and prestimulus alpha power around pupil maxima and minima. ***a***, Example of one participant's time course of the pupil size and detected maxima and minima marked with yellow and purple triangles, respectively; ***b*** and ***c***, Timecourse of ITC and alpha power, respectively, around pupil peaks and troughs; ***d***, cross-correlogram between ITC and alpha power associated with stimuli of the task-relevant modality: around pupil maxima (purple) and around pupil minima (yellow). Negative lags correspond to ITC leading alpha power, while positive lags correspond to alpha power leading ITC. Horizontal lines above and below the time courses indicate significant difference from zero based on permutation testing (*p* < 0.05).

As an exploratory analysis, we asked whether pupil-linked arousal may modulate the temporal relation between entrainment and alpha power. Specifically, we inspected entrainment and alpha power associated with stimuli of the task-relevant modality in periods centered around pupil maxima and minima (Fig. 5*a*; Montefusco-Siegmund et al., 2022). Although these maxima and minima roughly correspond to the extreme pupil bins in Figures 2 and 4, *a* and *b*, they are partly distinct in that they involve moments in which the derivative of the pupil signal, thought to track distinct changes in cortical state (Reimer et al., 2014; McGinley et al., 2015b), changes sign (e.g., from positive to negative around pupil peaks). We found that pupil maxima were preceded by an increase in entrainment, but this effect was transient and small (Fig. 5*b*). More interestingly, a large increase in prestimulus alpha was observed prior to pupil maxima and a large decrease prior to pupil minima (Fig. 5*c*). This indicates that changes in the electrophysiological signatures occurred prior to the changes in pupil-linked arousal. To investigate whether the changes in entrainment and alpha power associated with changes in arousal were linked, we calculated the cross-correlation between these signals around pupil maxima and minima. We found that the temporal relation between entrainment and alpha power was strongly modulated around pupil maxima and minima (Fig. 5*d*): there was a significant positive correlation between ITC and alpha power in time windows around pupil maxima and a significant negative correlation in time windows around pupil minima. Interestingly, while the positive correlation was transient, the negative correlation lasted for a prolonged time (∼18 s). Taken together, the results suggest that changes in ITC and alpha power are temporally related and that this relation is strongly modulated by arousal.

## Discussion

The goal of the current study was to unravel how variations in the brain’s internal state contribute to fluctuations in neural and behavioral measures of attention at slow timescales. We examined the impact of varying levels of pupil-linked arousal and alpha power on low-frequency phase entrainment. We found that increases in pupil size coincided with stronger entrainment of low-frequency oscillations to the task-relevant stimulus sequence. In contrast, increases in prestimulus alpha power coincided with a *decrease* in oscillatory phase entrainment to both the task-relevant and task-irrelevant stimulus sequences. Furthermore, we found that alpha power and ITC showed a significant correlation but only in the time windows around pupil maxima and minima, with the sign of the correlation depending strongly on whether pupil-linked arousal was high or low. These results suggest that variability in internal state modulates how the brain responds to external rhythmic stimulation and that arousal is a key factor coordinating the oscillatory mechanisms of the brain.

### Facilitatory effect of arousal on low-frequency entrainment

A key finding in our study was that increases in the pupil size coincided with a selective increase of low-frequency phase entrainment to the task-relevant stimulus sequence. While previous work on arousal-related effects on cortical function has largely focused on processing of single stimuli (either unpredictable or predictable), how arousal may benefit the processing of rhythmic stimulation has been less studied. Previous research has found that diminished phase entrainment is associated with lapses in attention and response accuracy (van den Brink et al., 2014; Lakatos et al., 2016). Our finding suggests that transient decreases in pupil-linked arousal may be one factor underlying these observations. Studies in animals suggest that pupil dilation is associated with changes in neuronal excitability and population activity (Reimer et al., 2014; Vinck et al., 2015; McGinley et al., 2015a), tuning the cortex into an active state (McGinley et al., 2015b). In addition, increased arousal is often associated with better task performance at low-to-intermediate arousal levels, while task performance decreases at very high levels of pupil-linked arousal, giving rise to an inverted-U relation between pupil-linked arousal and performance (van den Brink et al., 2016; Waschke et al., 2019; Beerendonk et al., 2023). Interestingly, Figure 4*a* shows decreased task-relevant ITC in the time windows with the largest baseline pupil size (bin 10). Our findings suggest that pupil-linked arousal, by shaping the brain’s internal state, affects how the brain processes task-relevant rhythmic stimuli.

Arousal-related neuromodulatory systems have been suggested to play a role in selective attention by setting the level of responsivity (i.e., gain) and excitability of the regions processing single stimuli that are salient or task-relevant (Dahl et al., 2022). In light of our results, one can speculate that while a “phasic” recruitment of neuromodulatory nuclei facilitates the processing of single stimuli, the “tonic” mode may be used for entrainment to sustained stimuli at low frequencies. While future studies are warranted to directly elucidate how neuromodulatory activity affects activity at the neuronal level during rhythmic stimulation, our results pose the arousal-related neuromodulatory systems as a highly flexible tool that can be recruited under different situations.

### Evidence for entrainment versus alpha processing modes

While larger pupil size was associated with a selective increase in low-frequency phase entrainment, larger alpha power was associated with an overall decrease in phase entrainment. These contrasting effects are surprising, given that alpha power and pupil size showed a strong positive relationship, a similar relationship with task performance (i.e., negative quadratic relationship with number of incorrect responses), as well as a clear temporal relationship (Fig. 5*c*): alpha power peaked before pupil maxima and reached a minimum before pupil minima—a similar pattern as in Montefusco-Siegmund et al. (2022). Moreover, both the pupil size and alpha desynchronization are thought to reflect activity of the noradrenergic system (Dahl et al., 2022). On the other hand, the partly dissociable effects of the pupil size (positively correlated with task-relevant phase entrainment; Fig. 4*a*) and posterior alpha power (negatively correlated with entrainment; Fig. 4*c*) seem consistent with previous work suggesting that spontaneous fluctuations in pupil size and alpha power jointly shape perception but modulate different aspects, i.e., perceptual sensitivity and perceptual bias, respectively (Waschke et al., 2019; Pilipenko and Samaha, 2024). Our study thus offers new evidence on a partial dissociation between these variables as mechanisms modulating attention to rhythmic stimulation.

A recent framework of oscillatory mechanisms proposes the existence of processing modes: an “entrainment mode,” in which sensory brain oscillatory mechanisms are engaged in processing rhythmic stimuli, and an “alpha-dominated mode,” in which the brain decouples from external stimulation (Zoefel and Van Rullen 2017). Our results support this framework in the sense that higher alpha power was related to decreases in entrainment to any (task-relevant and task-irrelevant) stimuli. While evidence for these modes was found in monkeys (Lakatos et al., 2016), here we found this effect also in humans. This is a novel result because it may reflect a general attentional mechanism comprising exogenous (i.e., led by task structure) and endogenous (led by internal state) processes. Furthermore, our results suggest that the shift between these modes is dependent on the level of arousal, as we discuss below.

### Arousal-dependent relation between prestimulus alpha power and low-frequency entrainment

Our exploratory analyses of the temporal relation between prestimulus alpha power, ITC, and pupil maxima/minima shed some light on the differential effects of pupil-linked arousal and alpha power: alpha power and task-relevant ITC showed a significant cross-correlation in the time windows around pupil maxima and minima, with the sign of the correlation depending strongly on whether pupil-linked arousal was high or low. We can only speculate why the coupling between entrainment and alpha power differed at the opposite extremes of arousal. One possibility is that the peaking of alpha power and correlation with ITC around pupil maxima reflected activity in brainstem arousal centers, which also drove the peaking of the pupil size. Indeed, activity in the locus ceruleus occurs shortly before pupil dilation (Joshi et al., 2016); pupil-linked arousal has generalized effects on the brain that are evident at slower time scales (Shine et al., 2016). On the other hand, the counter-relation around pupil minima may have reflected an inattention mechanism within the alpha band that emerged at low arousal. Indeed, it has been proposed that top–down and primary sensory circuits may contribute to the alpha band with opposing effects (Bollimunta et al., 2008). During low arousal, cortical effects on alpha power may signal basic sensory processes, which are dissociated from pupil-linked arousal effects (Pilipenko and Samaha, 2024). Lakatos et al. (2016) reported a slow anticorrelation in nonhuman primates between alpha power and ITC, as discussed above, and a recent study in humans reported a similar anticorrelation with entrainment to auditory stimuli (Kasten et al., 2024). Intracranial measurements in humans also showed a modulation of alpha power by the phase of delta entrainment (Gomez-Ramirez et al., 2011). In terms of mechanisms of selective attention, we speculate that arousal is related to higher entrainment and cognitive contributions to alpha power (Sadaghiani and Kleinschmidt, 2016; Clayton et al., 2018). A slow countermodulation between sensory alpha power and entrainment, rather than being sustained, may emerge during periods of low arousal. Given that the results in humans and nonhuman primates suggest the translational value of the findings discussed here, future research should replicate and further disentangle the relation between alpha power and ITC during periods of high and low pupil-linked arousal.

### Limitations and future directions

From a methodological perspective, it is hard to distinguish between true entrainment of ongoing neural oscillations and the consistent phase alignment caused by a sequence of stimulus-evoked potentials (Haegens and Golumbic 2018; van Diepen and Mazaheri, 2018; Helfrich et al., 2019). Nevertheless, mounting evidence shows that phase coherence persists during no-stimulus periods, suggesting that it is at least in part caused by neural entrainment (Zoefel et al., 2018; Bouwer et al., 2023), and intracranial studies in humans support the presence of widespread alignment of oscillations to rhythmic stimuli, not restricted to sensory areas (Besle et al., 2011; Gomez-Ramirez et al., 2011).

Previous studies have found within-trial phase–amplitude coupling between the delta phase of entrained oscillatory activity and alpha power over posterior brain regions (Wostmann et al., 2016; Wilson and Foxe, 2020). Here we were interested in fluctuations at slow time scales, similar to Lakatos et al. (2016), because they likely represent variation in the brain’s internal states. To compute the uniformity of phase angles across stimulus sequences, we used 8 s sliding time windows. The length of these time windows did not allow for subsecond temporal resolution, limiting our ability to study stimuli-specific relationships between entrainment and alpha power at various levels of arousal. Taken together, our results and previous studies suggest that internally and externally driven oscillatory mechanisms operate at different time scales.

As our study design was based on Lakatos et al. (2016), we did not strictly counterbalance the order of the attend auditory and attend visual blocks or the presentation frequencies of the auditory and visual stimulus sequences (i.e., resulting in more auditory trials for the computation of ITC). We could afford this because none of our key hypotheses (i.e., those corresponding to Fig. 4) involved a direct comparison between the two perceptual modalities; instead, we recoded the stimulus sequences as “task-relevant” and “task-irrelevant” and focused our analyses on that distinction, as in Lakatos et al. (2016). Although the difference in presentation frequency may have driven the modality effects in ITC, it is interesting that none of the relationships we observed between ITC, alpha power, pupil size, and behavior showed systematic differences between the auditory and visual modalities. This supports the assumption that our results reflect fluctuations in internal state rather than modality-specific effects.

Future work could assess the relationship between phase entrainment and another internal state variable: the slope of the 1/f shape of the power spectrum—i.e., the aperiodic (nonoscillatory) component of EEG activity (Waschke et al., 2021). This slope has been proposed to track the ratio between excitation and inhibition in underlying neural circuitry (Gao et al., 2017) and has been found to covary with spontaneous fluctuations in pupil-linked arousal (Pfeffer et al., 2022). In addition, alpha power estimates may be more reliable if aperiodic EEG is taken into account (Cunningham et al., 2023).

## Conclusions

Neural entrainment is an important instrument of selective attention (Schroeder and Lakatos, 2009). Here we reported delta-phase entrainment to both the visual and the auditory stimulus sequences, with stronger entrainment to the task-relevant modality than to the task-irrelevant modality. This is consistent with previous studies using bimodal stimulation (Besle et al., 2011; Lakatos et al., 2016). Most importantly, we found a significant linear relationship between pupil size and the degree to which entrainment tracked the task-relevant instead of the task-irrelevant stimulus sequence, indicating a facilitating effect of arousal on the perception of rhythmic stimulus sequences. We also found a coupling between alpha power and entrainment around pupil peaks and pupil troughs. Our results give clear indications of a differential modulation of brain oscillatory processes by arousal.

## Synthesis

Reviewing Editor: Christoph Michel, Universite de Geneve Decisions are customarily a result of the Reviewing Editor and the peer reviewers coming together and discussing their recommendations until a consensus is reached. When revisions are invited, a fact-based synthesis statement explaining their decision and outlining what is needed to prepare a revision will be listed below. The following reviewer(s) agreed to reveal their identity: Sebastiaan Mathôt, Niko Busch.

Both reviewers find the paper interesting and relevant. However, they both had difficulties to understand the analysis of the data. Several aspects were unclear to the reviewers and need to be addressed and explained.

I add both reviewer's comments and ask for a point-by-point response to each comment.

Reviewer 1:

The paper presents an experiment designed to investigate how pupil-linked arousal and alpha power in the EEG affect intertrial phase coherence (ITC) in a bimodal selective attention task. The results suggest that pupil-linked arousal mediates the relationship between alpha-power and ITC, and especially around pupil peaks and throughs, or high and low arousal-states, respectively. The chosen methods and analyses seem appropriate, and the paper is well-written. However, some points could be clarified on:

1. Why was the block order quasi-random and the same for all participants?

2. Was there a reason for always presenting the auditory stimuli at a higher frequency than the visual stimuli? Why was this not counterbalanced across participants?

3. In the pupillometry section it is mentioned that the exponential decay in the beginning of each block was regressed out of the pupil data. It would be useful to see some more reasoning for this. The decay is also a reflection of fluctuations in arousal, and as such could be theoretically meaningful, but is now disregarded in the analyses.

4. The description of which time windows were used for the different analyses is unclear, and it also seems that the pupil size, ITC, and frequency power data were analyzed in different ways that are difficult to compare. Specifically, and if we understand correctly, the pupil data was split into 2-second time windows, each of which reflecting the average of overlapping 8-second windows, over the continuous measurement period (not locked to the stimulus), while the EEG data was epoched into segments centered around the stimulus onset. More generally, this aspect of the analysis should be explained in more detail, possibly using a diagram. At present, it is difficult to assess whether the analysis procedure was valid.

5. Only hits and misses are mentioned in the statistics section: were there any false alarms? More generally, an analysis in terms of signal-detection theory (sensitivity and criterion) would be useful.

6. In the statistics section linear regression is reported as the chosen analysis to examine relationships between ITC, pupil size, prestimulus alpha power and task performance. Given that this is trial-level data that does not seem to be averaged, a regular linear regression would not be appropriate. Was a linear-mixed effects analysis performed instead? Or was the data aggregated before running a linear regression? More generally, more information should be provided about the specifics of the statistical analysis.

Minor comments::

1. In the description of Figure 1b; the intertrial coherence of visual trials is labeled ITCw rather than ITCv. The description also mentions 2-second steps but in-text the segments are reported to be 3 seconds long.

2. The room is reported to be dimly lit; what was the actual illuminance value?

3. What was the size (in degrees of visual angle) of the visual targets?

4. In the y-axis labels of Fig 4 ITC is labeled with a (z) but there is no in-text explanation of how it was normalized. Specifically, was this done for each participant separately?

5. X-axes for Fig. 5b, c, d are missing units.

Reviewer 2:

This study investigated the mutual relationships between different measures of arousal (pupil size) excitability (posterior alpha bad power) and stimulus-evoked neural activity (inter-trial phase coherence) in humans. The experiment used an audio-visual, inter-imodal attention task with rhythmic stimulation.

Although pupil size has been a known measure of arousal for several decades, there has been a recent surge of interest regarding its relationship with neuronal activity. Several recent studies have investigated the relationship between pupil size and electroencephalogram (EEG) or magnetoencephalography (MEG) activity in humans. The unique contribution of this study is that "neuronal activity" was quantified as inter-trial coherence in a paradigm with rhythmic stimulation, rather than as ongoing spontaneous EEG activity. ITC is an interesting and relevant variable because it is an established measure of stimulus-evoked activity that can reflect attentional deployment to a stimulus or sensory modality.

Overall, I found this study very interesting, and I am optimistic that it will be of substantial interest in this field of research. However, I admit that it is difficult to fully assess the quality of the method and results because I found the presentation of some of the analysis procedures very confusing. The methods need to be clarified to improve the understanding of the results and their interpretation before I can recommend this manuscript for publication.

# Major

## Description of methods needs clarification

I generally find the analysis pipeline and terminology hard to grasp. As a result, I was often not entirely sure how different parts of the analysis are related and how to interpret the results.

### Time-resolved data analysis

I am not sure I understand how the data were analyzed. To recap, the experiment consisted of 10 blocks, in which subjects were instructed to attend to either the visual or auditory stimuli. Both types of blocks used the same physical stimulation comprising a visual and an auditory stimulus stream. Each block was approximately 5 minutes long and comprised 265 visual and 340 auditory stimuli. How were pupil size, ITC and alpha power quantified in a time-resolved way?

The methods section specifies that "Then the data were split into Attend Auditory and Attend Visual conditions and epoched into 3-second segments centered on stimulus onset". I assume that "stimulus" refers to individual visual and auditory stimuli, meaning that this procedure resulted in 265 + 340 segments of 3 seconds per block (app. 30 minutes in total). Given that stimuli were presented at interleaved rhythms of 1.1 and 1.4 Hz, these 605 segments are strongly overlapping.

- What was the rationale and what makes this overlap meaningful?

- Why were these overlapping segments created before preprocessing was finished? Does ICA provide meaningful results when applied to these redundant data?

Data were analyzed with a "Morlet wavelet decomposition from 0.5 to 30 Hz with 30 logarithmically spaced steps."

- According to Matlab (logspace(log10(0.5), log10(30), 30)), the frequencies used in the wavelet analysis do not include the frequencies of stimulation (1.1 and 1.4 Hz). The nearest frequencies are 1.1664 and 1.3433, which are directly adjacent in this spacing. How were ITC and power quantified specifically at the frequencies of stimulation? For example, line 245 mentions that "strong ITC at the frequency corresponding to the task-relevant stimulus sequence (1.4 Hz for Attend Auditory, 1.1 Hz for Attend Visual), suggesting entrainment of EEG oscillations to the task-relevant stimulus rhythm."

- How long were the wavelet functions and what was the temporal/spectral precision (6)?

- Figure 3 shows ITC over a 3 second time window. How is this possible given that ITC was computed over 3 second segments? How did the analysis address the cone of influence, i.e. the time range that is potentially affected by edge-effect artifacts arising from areas where the wavelets extend beyond the edges of the segment.

Following this segmentation, "we took the 3-second segments mentioned above and computed for each 8-second time window the intertrial phase coherence (ITC), a measure of the consistency across trials of the phase angle at the time of stimulus onset".

- I am not sure what this means. How can the window (8 secs) be longer than the length of the segments (3 secs)?

- Why does figure 1 state that ITC was calculated in 8 seconds windows in 2 second steps?

- What exactly constitutes a "trial" in this experiment? I have a hunch that "trial" is synonymous for an individual visual or auditory stimulus, but I find this wording confusing. First, I think it is generally preferable to use a single technical term for a given concept. Second, a 'trial' typically refers to a single instance of stimulus presentation that requires a one-time execution of a specified perceptual or cognitive process, along with making (or withholding) a single behavioral response.

Alpha power was computed based on an FFT over a 500 ms long window "before the stimulus". " For the time window analyses, we computed the average alpha power value across all visual and (separately) auditory stimuli of which the onset latency fell within the 8-second window. "

- What does "the time window analysis" refer to exactly? Does this procedure also involve computing and averaging highly overlapping segments?

- Why were ITC and alpha power quantified using different methods? Both the wavelet and the FFT analysis yields a phase estimate from which ITC can be calculated and a power estimate.

"The analyses described above eventually yielded one pupil-size value, two ITC values and two alpha power values for each 8-second block: one for the task-relevant stimulus sequence (visual trials in the Attend Visual condition; auditory trials in the Attend Auditory condition) and one for the task-irrelevant stimulus sequence (visual trials in the Attend Auditory condition; auditory trials in the

Attend Visual condition)." I am not sure I understand this statement.

- First, it may be confusing because if I understand correctly, the statement following the colon does not actually refer to the one pupil size value.

- For ITC, I would have assumed that each 8 sec window yields 2 values: one at the frequency of the visual stream and one for the frequency of the auditory stream. But this is independent of the attention condition. Moreover, each 8 sec window only has a single attention condition, so how can the 2 ITC values for each 8 sec window refer to different attention conditions?

- Unlike ITC, alpha power is not related to a specific stimulation frequency, so how is it possible to get 2 alpha power values for each 8 sec window?

### Other issues

- A central part of the analysis tests for relationships between the relevant variables using binning (e.g. in Figure 2 and 4). The "exploratory" analysis then tests again for this relationship specifically by comparing data with minimal (troughs) and maximal (peaks) pupil size. How exactly is this different from the binning analysis? Aren't the minima and maxima roughly corresponding to extreme bins of the main analysis?

## Analysis and discussion should be expanded

- The experiment used an inter-modal attention task, in which either the visual or auditory stream was task-relevant. Many of the analyses collapse across the modality factor or recode the modality as task-relevant vs. task-irrelevant. I find this an oversight for several reasons:

- The manuscript discusses pupil size as a measure of arousal. However, the pupil is also part of the sensory apparatus of the visual, but not the auditory system, which leads to effects on peripheral encoding of visual, but not auditory stimuli (see 1). This may, in turn, yield differential effects of pupil size in visual vs. auditory attention tasks.

- Likewise, alpha-band power is discussed here as a measure of neuronal excitability. However, since the alpha rhythm is strongest over posterior, presumably visual cortex, it may also have a differential effect depending on the task-relevant sensory modality.

- Even where the modalities are considered separately, e.g. in the effect of pupil and alpha bins on task performance (Figure 2) , the manuscript reports only main effects, but not bin x modality interactions. Even if the interactions were not statistically significant, they should be reported. From visual inspection, it appears the effects of pupil bin on incorrect responses and of alpha bin on hits are both stronger for the visual modality.

- Most studies in this field have used only a single task modality or have used task-free resting state data, making this a unique opportunity for the present study. I therefore encourage the authors to pay more attention to modality-differences in the analysis and discussion.

- The discussion summarizes the findings as: "These results suggest that variability in internal state modulates how the brain responds to external rhythmic stimulation, and that arousal is a key factor coordinating the oscillatory mechanisms of the brain" (l. 320). I would appreciate if the discussion (and analysis) also addresses how arousal and excitability, i.e. internal brain state, influence selective attention, e.g. as indicated by the differential modulation of ITC for the relevant/irrelevant stimulus stream.

- I would appreciate a more elaborate discussion that discusses more in depth how the present results are related to the extant literature on arousal and excitability, e.g. as described in 2--5, 7--9. Are the findings consistent? Are any result surprising? What does the present study bring to the table that has not been addressed by previous studies on this topic?

- The analysis is focused on pupil size and alpha power as measures of "internal brain state". However, it is becoming increasingly clear the another relevant aspect of brain state, and specifically of balance of neuronal excitation/inhibition, is the non-rhythmic 1/f shape of the power spectrum (e.g. 2--5). When ignored, fluctuations in non-rhythmic patterns can confound effects of narrow-band power, such as alpha activity. Therefore, I encourage the authors to consider also this measure of excitation/inhibition.

- How did pupil size, alpha, and ITC respond to appearance of target stimuli, and could such responses account for some of the temporal correlation between these measures?

# Minor

- This may just be personal preference, but I find the terms "peak" and "trough" for the time course of pupil size a bit misleading. To me, these terms refer to phases of a wave-like, oscillatory signal pattern, which the pupil signal clearly is not. Why not simply "maxima" and "minima"?

- In what sense was the cross-correlation analysis "exploratory"? Were the other analyses pre-registerd?

- Raw pupil size showed an exponential decrease at the start of each block, but these effects were removed from the data statistically. Please elaborate on the rationale. Why do you suppose pupil size change so much? If pupil size is a relevant measure of arousal, why isn't its entire range of variations relevant to analyze?

- "Visual stimuli were made isoluminant with the grey screen background (RGB 167 167 167) using the SHINEcolor toolbox"; please elaborate.

- "Bad channels were removed, with the rejection threshold for channel correlation set to the default of 0.8 and the artifact subspace reconstruction parameter set to 10."; please explain what these parameters and procedurs mean.

- "We analyzed the variables ITC and prestimulus alpha power in segments of 10 windows around peaks and troughs of the pupil timecourse" ; how long were these windows?

- Please elaborate how the cross-correlations were computed.

- "the temporal relation between entrainment and alpha power was strongly modulated around zero-crossings of the pupil derivative"; what do "zero crossings" and "pupil derivative" refer to? The emthods should explain these terms and how they are computed.

- Will the data and code be made publicly available online?

# References

- (1) Mathôt, S., Berberyan, H., Büchel, P., Ruuskanen, V., Vilotijević, A., &Kruijne, W. (2023). Effects of pupil size as manipulated through ipRGC activation on visual processing. *Neuroimage*, *283*, 120420.

- (2) Waschke, L., Tune, S., &Obleser, J. (2019). Local cortical desynchronization and pupil-linked arousal differentially shape brain states for optimal sensory performance. *Elife*, *8*, e51501.

- (3) Waschke, L., Donoghue, T., Fiedler, L., Smith, S., Garrett, D. D., Voytek, B., &Obleser, J. (2021). Modality-specific tracking of attention and sensory statistics in the human electrophysiological spectral exponent. *Elife*, *10*, e70068.

- (4) Pfeffer, T., Keitel, C., Kluger, D. S., Keitel, A., Russmann, A., Thut, G., ... &Gross, J. (2022). Coupling of pupil-and neuronal population dynamics reveals diverse influences of arousal on cortical processing. *elife*, *11*, e71890.

- (5) Cunningham, E., Zimnicki, C., &Beck, D. M. (2023). The Influence of Prestimulus 1/f-Like versus Alpha-Band Activity on Subjective Awareness of Auditory and Visual Stimuli. *Journal of Neuroscience*, *43*(37), 6447-6459.

- (6) Cohen, M. X. (2019). A better way to define and describe Morlet wavelets for time-frequency analysis. *NeuroImage*, *199*, 81-86.

- (7) Dahl, M. J., Mather, M., &Werkle-Bergner, M. (2022). Noradrenergic modulation of rhythmic neural activity shapes selective attention. *Trends in cognitive sciences*, *26*(1), 38-52.

- (8) Radetz, A., &Siegel, M. (2022). Spectral fingerprints of cortical neuromodulation. *Journal of Neuroscience*, *42*(18), 3836-3846.

- (9) Pilipenko, A., &Samaha, J. (2024). Double dissociation of spontaneous alpha-band activity and pupil-linked arousal on additive and multiplicative perceptual gain. *Journal of Neuroscience*, *44*(19).
