# Supplementary figures and images for: Strength of Low-Frequency EEG Phase Entrainment to External Stimuli Is Associated with Fluctuations in the Brain's Internal State
Source: eNeuro. 2025 Jan 17;12(1):ENEURO.0064-24.2024. doi: 10.1523/ENEURO.0064-24.2024 (PMC11772043; doi:10.1523/ENEURO.0064-24.2024)

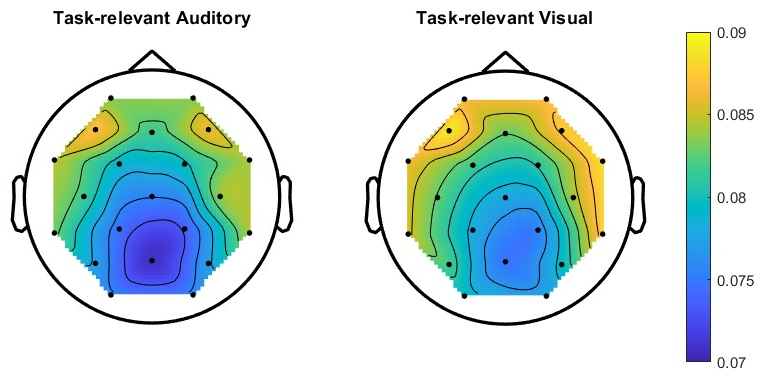

Supplement: Figure 3-1 — Topoplots of pre-stimulus alpha power distribution for task-relevant stimuli. Download Figure 3-1, TIF file. [file eneuro-12-ENEURO.0064-24.2024-s001.tif]

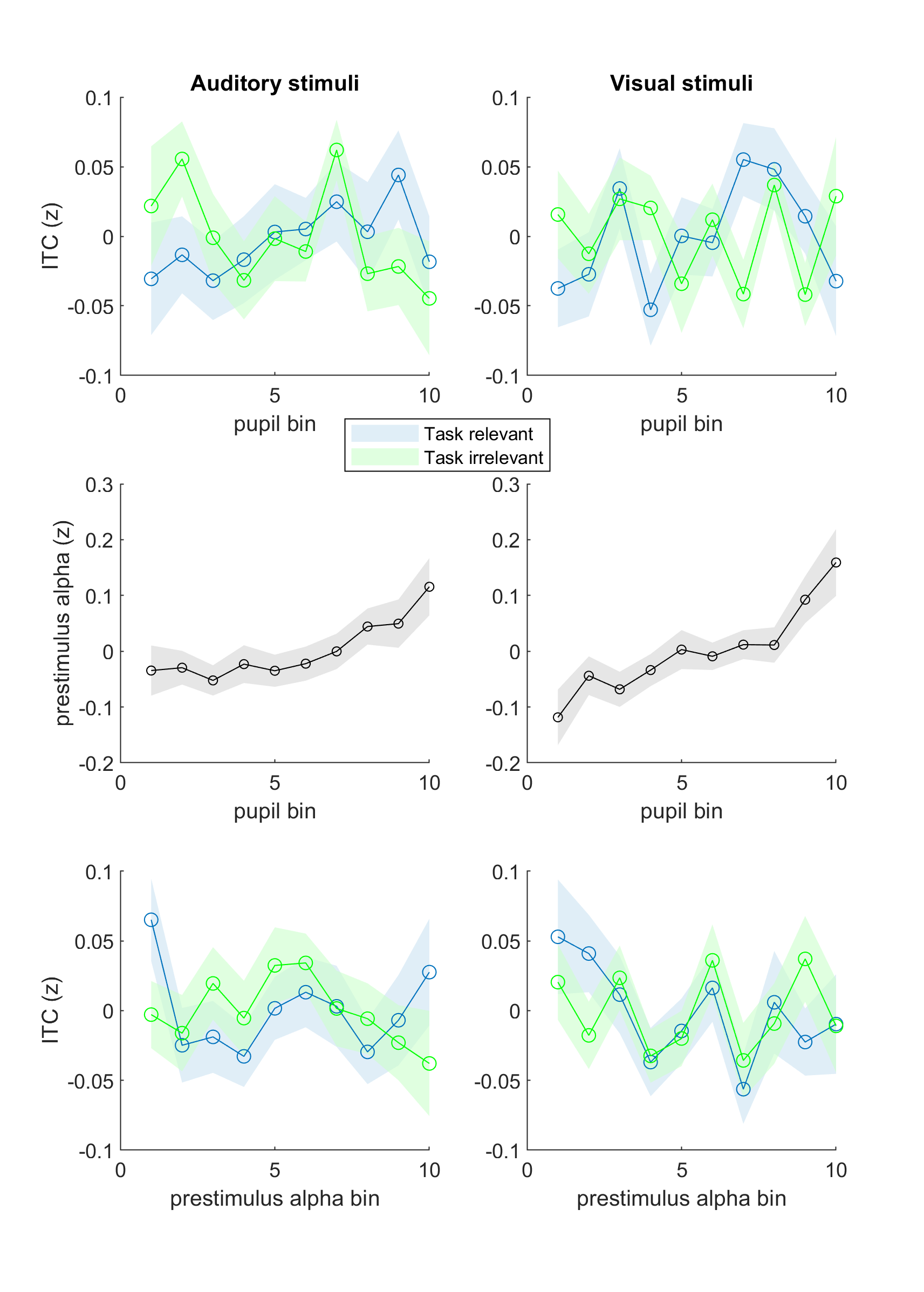

Supplement: Figure 4-1 — Paired relationships between pupil size, ITC and alpha power separating by stimulus modality. Left column: auditory stimuli in Attend Auditory blocks (i.e., auditory stimuli were task-relevant, blue) and Attend Visual blocks (i.e., auditory stimuli were task-irrelevant, green). Right column: visual stimuli in Attend Visual blocks (i.e., visual stimuli were task-relevant, blue) and Attend Auditory blocks (i.e., visual stimuli were task-irrelevant, green). Top row: pupil size and ITC; Middle row: pupil size and prestimulus alpha power; bottom row: prestimulus alpha power and ITC. Download Figure 4-1, TIF file. [file eneuro-12-ENEURO.0064-24.2024-s002.tif]

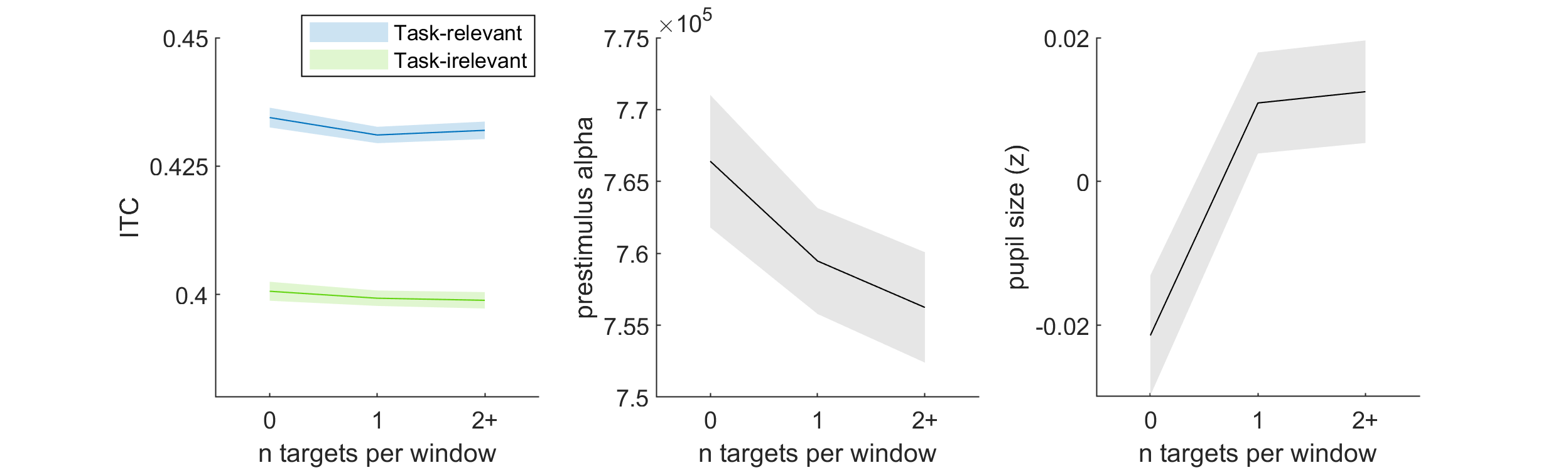

Supplement: Figure 4-2 — Effect of number of targets on pupil size, ITC and alpha power. We examined if the relationships between our four key dependent variables might be confounded by enlarged evoked EEG or pupil responses to infrequent target stimuli. The temporal overlap between the auditory and visual sequences made it hard to estimate single-trial evoked responses, but we used a more indirect way to address the issue: We assessed if ITC, and average alpha power and pupil size in a given window depended on the number of targets in that window. If two variables were each dependent on the number of targets, then their relationship might be due to this confound rather than slow co-fluctuations in internal state. We found no effect of number of targets (0, 1 or 2+) on ITC in the task-relevant (F = 1.35, p = 0.25) or task-irrelevant modality (F = 0.99, p = 0.41, Fig. 4.2). We found no effect of number of targets on prestimulus alpha (F = 1.47, p = 0.228). We found an effect of number of targets on pupil size (F = 2.91, p = 0.020), with increasing pupil size for increasing numbers of targets. The absence of an effect on ITC and alpha power suggests that number of targets cannot be driving the significant relation between this variable and pupil size in Fig. 4.b. Download Figure 4-2, TIF file. [file eneuro-12-ENEURO.0064-24.2024-s003.tif]
